# An Evaluation of the Usability and Feasibility of the 50K4Life Mobile App for Delivering Walking Challenges to Public School Administrative Employees: Beta Testing Study

**DOI:** 10.2196/80341

**Published:** 2026-06-25

**Authors:** Jennifer J Salinas, Valery Giselle Lopez, Jennifer L Gay, Susan W Buchholz, Eden Hernandez Robles, Larissa Meza, Angelica Juarez, Maysa Sapargeldiyeva, Deborah Parra-Medina, Zenong Yin

**Affiliations:** 1 Department of Social Work College of Health Sciences University of Texas at El Paso El Paso, TX United States; 2 Health Promotion & Behavior College of Public Health University of Georgia Athens Georgia; 3 College of Nursing MSU College of Nursing East Lansing, MI United States; 4 Worden School of Social Services Our Lady of the Lakes University San Antonio, TX United States; 5 Department of Public Health College for Health Community and Policy The University of Texas at San Antonio San Antonio, TX United States; 6 Department of Family Medicine School of Medicine CU Anschutz Medical Campus Aurora, CO United States

**Keywords:** digital health, mobile health, mHealth, physical activity, workplace, Mexican Americans, app development

## Abstract

**Background:**

Mobile health apps show promise for delivering physical activity interventions, but uptake remains low due to usability barriers. Beta testing is essential to optimize user experience before full implementation.

**Objective:**

This study aimed to evaluate the usability, acceptability, and feasibility of the 50K4Life mobile app prototype for delivering a 2-week walking challenge to public school administrative employees.

**Methods:**

Following the Integrate, Design, Assess, and Share framework, we conducted a single-group beta test with 12 public school administrative employees in El Paso County, Texas. Participants used the 50K4Life app built on the Pathverse platform for a 2-week walking challenge. Data collection included acceptability surveys, satisfaction questionnaires, app use metrics, and qualitative debriefing sessions.

**Results:**

All 12 participants completed the walking challenge. Acceptability was high for app design (n=12, 91.7%), layout (n=9, 75%), and battery impact (n=10, 83.3% reported no issues). However, participants experienced difficulties with navigation (n=7, 58.3%), delays in updating step counts (n=8, 66.7%), and completion of assigned tasks (n=5, 41.7% could not locate all features). App use was high: 100% (n=12) accessed the leaderboard and walking challenge page, and 91.6% (n=11) synced step data and set step goals. Results from participants’ responses in the surveys and feedback from debrief interviews identified needs for improving user engagement features, synchronization, and visual appeal.

**Conclusions:**

The application of a participatory approach and Integrate, Design, Assess, and Share framework yielded valuable insights into the acceptability of the 50K4Life app reported by this study cohort and potential for enhanced features in *real-world* use contexts.

## Introduction

Mobile health (mHealth) technologies refer to mobile devices such as smartphones and wearable devices, SMS text messaging, and computer applications for mobile devices that are useful tools for delivering and supporting evidence-based self-management programs to improve health behaviors and reduce chronic disease risk [1,2]. Research has shown that mHealth interventions are effective in improving lifestyle behaviors [3-6], averting medical emergencies [7,8], contributing to disease management [2], offering support to caregiving [9], and supporting mental health [10] across the population groups. The emerging multi-billion-dollar smartphone industry has facilitated widespread and real-time access to evidence-based health practices and therefore may have a broad impact on the general population’s health. In 2021, a total of 85% of America adults reported owing a smartphone [11]. The popularity of smartphones, along with advances in smartphone technology and 5G internet, has promoted the scalability of smartphones and apps for the uptake of chronic disease self-management practices through self-monitoring of lifestyle behaviors [2,12].

Recent research has examined barriers that hinder adopting mHealth interventions, focusing on issues related to poor user experience in accessing and navigating mHealth apps, support for problem solving, and browsing the content of didactic education [13-16]. These issues can influence user’s engagement in mHealth interventions and reduce the effectiveness on targeted outcomes [13]. Moreover, inflexibility in tailoring program content and format for users’ needs [17,18], inappropriate program context [19], lack of personalized content, communication [14,20], and incompatibility with users’ preferred devices [21] make the use of mHealth apps cumbersome in community and clinical settings. These issues, along with not leveraging mHealth apps’ interactive capabilities and not incorporating behavior change techniques [15,18,22,23] can disengage users’ regular and sustained engagement in the intervention activities to achieve significant health outcomes. Therefore, it is critical to conduct studies that are intended to understand these barriers and adapt delivery approaches to improve usability and acceptability (ie, beta tests) to fully realize their promise in health promotion [24-26]. A recent systematic review recommended an iterative process to test the reliability, usability, and safety of new mHealth apps to improving access, efficiency, and user experience before deployment in the real-world setting [27]. mHealth apps are widely used to promote participation in physical activity, including walking [28,29]. Walking has been recommended as a low-cost, effective approach to overcome barriers to engaging in daily physical activity for workplace [30] and in community settings [31]. Walking challenge is an evidence-based practice using *steps* tracked through a tracking device, such as smart watches, to increase physical activity using behavioral techniques such as time restriction, goal achievement, competition, reward, and teamwork [32,33]. However, poor user experience, inadequate customization to user preference, and lack of embedding of evidence-based strategies has dampened participants’ engagement in app-based walking programs [34-36].

This study reports on the user acceptability of content and design, usability, user satisfaction, and functionality of a prototype mHealth app developed with the Pathverse platform (version 2.14.49). The app was developed to deliver a walking promotion intervention, “50,000 Steps for Life (50K4Life)” to public school employees in El Paso County, Texas, based on our prior work in promoting daily physical activity through walking challenge campaigns in the El Paso community [37]. 50k4Life is a sequential multiple assignment randomized trial to promote walking among public school teachers [38]. The 50K4Life app will deliver walking challenges using evidence-based strategies for workplace health promotion [14,39] over the course of the school year. The app will be used to monitor participant’s individual and school’s progress in the walking challenges through the app-based tracker and leaderboard as well as implement evidence-based practices to promote walking at school and home. Although many walking promotion apps have features such as step tracking and self-monitoring [40,41], research is sparse on how to customize apps to deliver tailored walking challenges and evidence-based practices specifically designed for 50K4Life [37]. Moreover, given the limited evidence of the feasibility and effectiveness of app-delivered walking promotion programs in the United States [34,42], the findings from this beta test will shape next steps in their dissemination and uptake for community and clinical use. In addition, the information obtained will be used to refine the app components in a pilot study and to prepare for the full trial as part of intervention delivery.

## Methods

### Study Design

This study addressed the first 2 stages of the Integrate, Design, Assess, and Share (IDEAS) framework in designing digital health interventions to promote changes in health behaviors [43]. The IDEAS framework allowed the study team to systematically assess the study needs and incorporate evidence-based strategies from the literature and our previous work in designing a user-friendly, multipurpose app delivering the 50K4Life intervention to public school employees [41,44] using the Pathverse platform [45]. Pathverse is a no-code mobile app builder that can customize app features and functions to deliver and monitor intervention content from the researcher portal with full autonomy. Specifically, Pathverse offers the features and functions to deliver program content (eg, program announcement, access to health lessons, videos, and resources), embed behavioral change tools (eg, self-monitoring and goal setting), gather input from participants (eg, quizzes and surveys), communicate with participants (push notifications and community forum), and collect use data of features and functions for monitoring and evaluation. The app is available in English and Spanish for iOS in the Apple App Store and for Android in the Google Play Store.

In stage 1, Integrate, the multidisciplinary research team collaborated with the 50K4Life study community advisory board comprised of representatives of school administration, instruction and curriculum and school wellness program personnel, and stakeholders from communities to discover and define the background target users and create a profile, that is, public school employees of diverse socioeconomic background, and to specify the targeted behavioral outcomes of the 50K4Life intervention that will be accommodated in the app. The research team shared the delivery strategies and expected outcomes of the 50K4Life intervention and available features and functionalities of the Pathverse platform with community advisory board members, and received initial input on the layout of the 50K4Life app to meet the study’s needs in a workplace setting. In addition, the research team worked closely with Pathverse engineers to understand the app’s capabilities, delivering the intervention in alignment with the socioecological model [46], the behavioral change theory guiding the design of the 50K4Life intervention, and to assure appropriate targeting of individual, interpersonal, and organizational and environmental factors influencing walking behavior at workplace and home [44].

In stage 2, Design, the study team translated the information gathered in stage 1 to develop a prototype app, including features and functionalities to deliver various aspects of the walking challenge (Table 1). The prototype app was evaluated in a beta test with a small group of public school employees who were asked to perform a series of tasks that provided data on access, use, and satisfaction of features and functions in the app. The participants also provided feedback on the user experience and recommendations to improve user experience and optimize app features and functions. Finally, the study team incorporated the results from the beta test and built a minimum viable app for a pilot on its usability and feasibility in stage 3 of IDEAS, Assess. Results of stage 3, the pilot study, were not reported in this manuscript.

**Table 1:** Features and functions of the 50K4Life app and tasks performed by the study participants.

Feature and function of the app	Task	Expected use
50K4Life app	Participants downloaded the 50K4Life app to a smartphone and linked the app to an activity tracker	One time at the beginning of beta test
50K4Life walking challenge home page: announcing the walking challenge of 50,000 steps a week for 2 weeks	Participants viewed the challenge goals and instructions on the home page	At least one time
Syncing data to the 50K4Life app: uploading step counts to the leaderboard	Participants uploaded data from an activity tracker (eg, Apple Watch and Fitbit) to the 50K4Life app	At least one time a week
Self-monitoring on the 50K4Life dashboard	Participants viewed their own and team members’ step counts to engage in self-monitoring and competition with peers	At least one time a week
Goal-setting tool to set walk goals	Participants set and monitored walking goal	At least one time a week
Automated push notifications to participants	Participants received push notifications of motivational messages and reminders related to walking challenge and data collection	Review 4 notifications a week
Intervention content (1 health education lesson and walking videos) supporting the 50K4Life walking challenge	Participants accessed and viewed health education lesson and resources	One time
Community forum	Participants made posts in the forum to share experiences and engage others in the 50K4Life walking challenge	One time a week
Participant feedback	Participants completed a weekly injury survey and health lesson completion survey to provide feedback to the study team	One time a week for injury survey and one time for health lesson survey

### Setting

Participants were recruited from 2 public school districts in El Paso County, Texas. El Paso residents are disproportionately burdened by health conditions associated with inadequate physical activity engagement [47]. Currently, 1 in 3 El Paso residents did not engage in any physical activity in the past month [48]. This population experiences earlier onset and poorer control of cardiometabolic conditions such as diabetes, hypertension, and fatty liver disease and lives more years with disabilities requiring assistance from family members or skilled nursing care [47]. Regular engagement in physical activity, including walking, can delay and possibly prevent the sequelae of disease, disability, and poor quality of life. Nevertheless, innovative physical activity programs widely used in other regions of the United States have not been implemented in this region despite their evidence-based effectiveness.

### Walking Challenge

During the app-based walking challenge, participants were asked to walk as many steps as they could each week for 2 weeks. Participants were able to monitor their personal, teammates’ and overall team’s step totals through the app’s leaderboard. The team with the most steps after 2 weeks was deemed the winner of the challenge. The winning team received the 50K4Life program and school district merchandise.

### Recruitment and Enrollment

Participants were recruited from the administration departments of 2 school districts. The administrative staff were tied to school instruction or services (eg, physical education, cafeteria, and custodial) and at some point in their career had worked on a school campus and therefore had insight into the feasibility and usability for school-based participants in the full trial. Inclusion criteria were employment in the administrative offices, aged ≥18 years, not pregnant, and owner of a smartphone. Individuals were excluded if they had a mobility issue that prevented them from fully participating in a 2-week-long walking challenge. Participants were identified by a designated school liaison, and contact information was given to the study staff. Study staff then contacted potential participants via email, inviting them to participate in the study. Once a potential participant was identified, an in-person appointment was arranged where informed consent and study orientation were conducted. A total of 12 participants were enrolled in the study.

### Beta Test Procedure

The beta test procedure included the following: (1) participants were tasked with activities related to using the 50K4Life app, such as finding information, tracking steps, completing educational modules, and other activities related to the study (Table 1); (2) participants took part in a 2-week-long walking challenge and tracked their progress through the 50K4Life app; (3) participants were sent a link to a survey in Research Electronic Data Capture (REDCap; Vanderbilt University) at the end of the 2 week walking challenge; and (4) after completing the walking challenge, participants took part in a debriefing session.

### Ethical Considerations

The study protocol was reviewed and approved by the institutional review board at the University of Texas at El Paso (protocol number 2089761). Written informed consent was obtained from all participants in either English or Spanish, depending on each participant’s preferred language. Participants were provided with a copy of the consent form, which included detailed information about the study, data collection, and their rights as participants. Data collection training for all study staff emphasized the importance of protecting participants’ privacy and maintaining confidentiality of data. Data collection, management, and quality assurance for this study were facilitated using REDCap electronic data capture tools hosted at the University of Texas at El Paso. REDCap is a Health Insurance Portability and Accountability Act—a secure data management system that ensures effective data collection, storage, retrieval, and quality control. As this was a walking challenge where participants were able to view each other’s steps and identities, an effort was made to provide options for participants to take part without using names or other identifiers. First, participants were given the option to create aliases when they registered with the 50K4Life app. The alias appeared on the leaderboard instead of their name or email address. The alias option was also offered during the debriefing sessions to protect confidentiality. Additionally, unique identification numbers were assigned to each participant and used to link all data. Once transcripts from debriefing sessions were cleaned and verified, aliases were removed, and initials were used instead. Participants were sent a US $25 gift card upon completing the debriefing session.

### Study Data

The primary outcomes of the study were app functionality, usability, and acceptability. Data were collected through surveys, interviews during debriefing sessions, and use data of the 50K4Life app.

#### Survey Description

A survey was emailed to each participant via REDCap and completed at the end of the second week of the walking challenge. Surveys took 10 to 15 minutes to complete and were available in both English and Spanish.

#### Demographics

Participants self-reported age, gender, occupation, role in the school district, and department.

#### Participant Experience With the App

Participants were asked 16 questions (yes or no) about their experience related to the acceptability and feasibility of features and functionality in the 50K4Life app. These questions ranged from locating the 50K4Life app features to accessibility and impact on cellphone battery life.

#### Participant Satisfaction With the App

There were 11 Likert-scale questions (1=strongly disagree, 4=strongly agree) asking participants to report their satisfaction and user experience with the features and functionality of the 50K4Life app. Questions ranged from ease of navigation to design and layout. Questions were developed by study staff and included questions that were determined by consensus.

#### In-App Surveys

To test the survey capability in the 50K4Life app, participants were asked to complete an embedded t3-question survey each week. The survey gathered information on incidence of injury and perceived environment at home and at work to achieve their step goals. Participants received push notifications alerting them to the survey and deadline for completion.

#### Use Data

Access to features and functions in the 50K4Life app was gathered using the app’s analytics tools which tracked the number of times participants had used each feature and function, and the amount of time spent per use. The app did not have the capability to track whether participants opened the push notifications.

#### Debriefing Sessions

A total of 4 debriefing sessions, each lasting 30 to 60 minutes, were conducted via Zoom (version 7.0.5) by a PhD-trained facilitator. The facilitator used a semistructured interview guide that included 5 topic areas: app likes, app dislikes, changes they would make, aspects that make the app challenging to use, and the app’s potential for walking motivation. The goal of the session was to capture feedback from participants regarding their experiences with the 50K4Life app. All sessions were recorded and transcribed using Zoom’s built-in video and transcription tools to facilitate analysis. Additionally, the notes taken by the 2 notetakers were incorporated into the transcripts to ensure the capture of key details that might not have been fully transcribed by the software, such as contextual information. Each debriefing session included 2 to 4 participants, along with 2 notetakers and a bilingual facilitator. All participants were provided with the option to join a debriefing session in either English or Spanish. Despite this option being available, all participants opted for the English-language sessions.

### Data Analysis

Participant survey responses were tabulated to check for validity and missingness. Means and SDs for continuous variables, and frequency and percentage for categorical variables were calculated.

Transcriptions from the debriefing sessions were then proofread for accuracy, formatting, and clarity. The study staff identified and extracted the main discussion points from each debriefing session. Themes were organized inductively into likes, dislikes, and suggestions for improvement. Likes were 50K4Life app aspects that participants found appealing, helpful, or motivating. Dislikes were areas of the app that caused frustration or dissatisfaction among users. Finally, suggestions for improvement were ideas and recommendations for enhancing the app’s functionality, user interface, or overall experience.

## Results

### Participant Characteristics

The beta test was conducted with 12 participants with diverse administrative roles and experiences, as well as an understanding of employees in public schools. A total of 3 participants withdrew from the walking challenge before completing it due to scheduling conflicts (n=2) and technical difficulties (n=1). A total of 7 participants used a study-issued Fitbit tracker, while the rest used either a personally owned Apple Watch or a Samsung Galaxy Watch. All participants completed the 2-week walking challenge and posttest assessment. Table 2 shows the participant demographics.

**Table 2:** Study participant demographics and types of activity trackers used in the beta test (N=12).

		Values
Age (years), mean (SD)		39 (10.7)
Sex, n (%)		
	Male	4 (33.3)
	Female	8 (66.7)
School district administrative role, n (%)		
	Business or accounting	2 (16.7)
	Human resources	3 (25)
	Athletics	1 (8.3)
	Curriculum instruction	1 (8.3)
	Nursing	1 (8.3)
	Security	1 (8.3)
	Food service	1 (8.3)
	Federal programs	1 (8.3)
	Not specified	1 (8.3)
Activity tracker type, n (%)		
	Fitbit inspire 3 health and fitness tracker	7 (58.3)
	Apple Watch	2 (16.7)
	Samsung Galaxy Watch	3 (25)

### Acceptability of Content and Design

Participants had mixed responses regarding the acceptability of features and functions in their experience with the 50K4Life app (Table 3). Acceptability was high for overall design and options in the app, access to the app, the app’s effect on battery life, the smoothness and operability of the app’s functions, the layout of features and functions in the app, and the app’s visual appearance (color scheme and fonts). The participants reported negative experiences navigating the app, viewing or accessing information display (step count updates), syncing the app with tracker and/or phone (requiring third-party apps to sync data), using certain features and functions to complete tasks, and enjoying the app’s features (lacking features engaging and motivating participants).

**Table 3:** Participant survey responses regarding the acceptability of the 50K4Life app features, functions, and operations (N=12).

			Yes, n (%)
Participant responses on the 50K4Life app features and functions			
	“Can you locate everything in the app?”	5 (41.7)	
	“Can you describe what every option does in the app?”	2 (16.7)	
	“Did you understand the layout and organization of the app’s features and content?”	9 (75)	
	“Did the color scheme and font selection contribute to a pleasant user experience?”	11 (91.7)	
	“Were there any aspects of the app’s design that you found distracting or unappealing?”	3 (25)	
	“Does the app have designs or elements that make this app enjoyable?”	8 (66.7)	
	“Were interactive elements (buttons, links, videos, etc) accessible to identify and use?”	5 (41.7)	
App operations			
	“Did you experience any delays while using the app?”	8 (66.7)	
	“Do you have or need accessibility requirements or preferences?”	0 (0)	
	“Does the app perform smoothly without any glitches or crashes?”	1 (8.33)	
	“Were you able to complete the tasks you attempted within the app?”	7 (58.3)	
	“Were you able to use all app features regardless of any accessibility needs you might have?”	4 (33.3)	
	“Do you notice any significant impact on your battery life while operating the app?”	2 (16.7)	
	“Did you encounter any difficulties or obstacles while trying to complete tasks?”	9 (75)	
	“Is the app compatible with your watch or tracker?”	7 (58.3)	
	“Is the app compatible with your phone?”	12 (100)	

### Ease of Use and Satisfaction

Table 4 shows the participant’s satisfaction with the 50K4Life app. With scores >3 on 11 questions, participants were satisfied with the app’s features and functions. The lowest scores were associated with navigation, viewing step counts, and app layout.

**Table 4:** Participant satisfaction with the 50K4Life app features and functions (N=12).

To what extent do you agree that... (1=strongly disagree and 4=strongly agree)	Values, mean (SD)	Agree or strongly agree, n (%)
“Is the app easy to navigate?”	3.2 (0.75)	4 (33.3)
“Are the menus and icons easy to access?”	3.3 (0.86)	4 (33.3)
“It was easy to access your walking data.”	3.1 (0.72)	3 (25)
“It was easy to edit your walking data.”	3.2 (0.54)	9 (75)
“You had a great experience with the app.”	3.3 (0.77)	10 (83.3)
“It was effortless to complete tasks on the app.”	3.3 (0.60)	8 (66.7)
“The app’s design and layout are great.”	3.2 (0.75)	8 (66.7)
“The app responded quickly to your interactions.”	3.5 (0.63)	5 (41.7)
“You encounter very little accessibility issues when using the app.”	3.4 (0.72)	7 (58.3)
“You did not need technical support for this app.”	3.4 (0.96)	7 (58.3)
“Finding what you were looking for in the app was very easy.”	3.3 (0.68)	9 (75)

### Usability of Features and Functions

Table 5 shows the usability data from participants’ access to functions and content in the 50K4Life app. The participants were instructed to use the features and functions to receive study-related information (eg, instructions for walking challenge and notifications from the study), access educational content or resources (eg, health education lessons), or perform a function (eg, setting walking goals and completing a survey) during the walking challenge. Except for using the survey function, the usability rate was ≥75%, indicating participants met the expected level of engagement (Table 1). The Pathverse platform cannot track if participants viewed the notifications sent by the 50K4Life app.

**Table 5:** App feature use during the 2-week walking challenge (N=12).

Features in app	Participants who accessed the feature	
	Values, n (%)	Values, mean (SD)
Walking challenge page	12 (100)	14.5 (10.4)
Leaderboard	12 (100)	11.92 (9.9)
Weekly syncing step counts	11 (91.6)	1.4 (0.7)
Health lesson 1: walking for health	11 (91.6)	3.58 (3.1)
Health lesson completion survey	8 (66.6)	2.17 (2.3)
Weekly injury survey	5 (41.6)	0.5 (0.7)
Setting walking goal	11 (91.6)	4 (3.7)
Walking videos	10 (83.3)	2.25 (2.4)
Community forum	9 (75)	4.08 (4.2)

### Debriefing Session Results

Overall, the participants reported that the app was easy to navigate and access, and the layout was organized and simple. The participants found that the leaderboard created a competitive environment that motivated and engaged them in the walking challenge. In addition, participants liked the step tracking feature, which used synced step counts from activity trackers, and found it effective at keeping them focused on the walking challenge. By contrast, participants reported difficulties and concerns that negatively affected their experience with the app during the beta test. In Table 6, we organized the issues and challenges reported by the participants into 5 problem areas and summarized the suggestions from participants to address these issues in the 50K4Life app. A quote from a participant is also included for each theme. These themes and suggestions provided additional guidance for refining the 50K4Life app.

**Table 6:** Themes and suggestions derived from participant debrief interviews.

Problem areas	Issues underlying the problem area	Potential solutions
Lack of user engagement features (“The app itself didn’t motivate me. It was more about the challenge itself.”)	Interactive features: messaging within groups, creating avatars, and earning rewards for meeting goals Community engagement elements: group challenges, leaderboards, and social interactions	Create gamifying elements (scavenger hunts or photo challenges) to encourage more physical activity Create a leaderboard for team competition Incorporate community forum into app for social engagement
Lack of customization and visual appeal (“We get so lost in work we are just focused on work. So the little things like our phone rings or messages pop up get our attention.”)	Features in the 50K4Life app lacked attractive color schemes or a personalized interface	Offer more customization options Add wallpaper or stickers to improve visual appearance and make it engaging for participants who achieve goals
Syncing problems (“The app would not sync with my watch. My steps were on my watch but not on the app.”)	Syncing difficulties between activity trackers and Pathverse server that prevented showing step counts in real time	Ensure seamless integration with fitness devices Improve the automatic synchronization of data from fitness devices to the app
Cumbersome app setup (“I would have given up and said, forget this. I don’t have time to deal with the watch and three different apps.”)	Participants needed to download multiple third-party apps to their smartphones to sync step count data to the 50K4Life app	Include frequently asked questions and support resources within the app to assist users with common issues during program initiation and during the walking challenge
Inconsistent delivery of push notifications (“I never even got that notification on the app, even though my notifications were turned on for that specific app. I don’t know. I just think systematically it could have been better.”)	Irregular and untimely delivery schedule of notifications reduced the value of the notifications	Implement more frequent and motivating notifications to remind users of their goals and progress

### Changes in Step Counts

**Figure 1:**
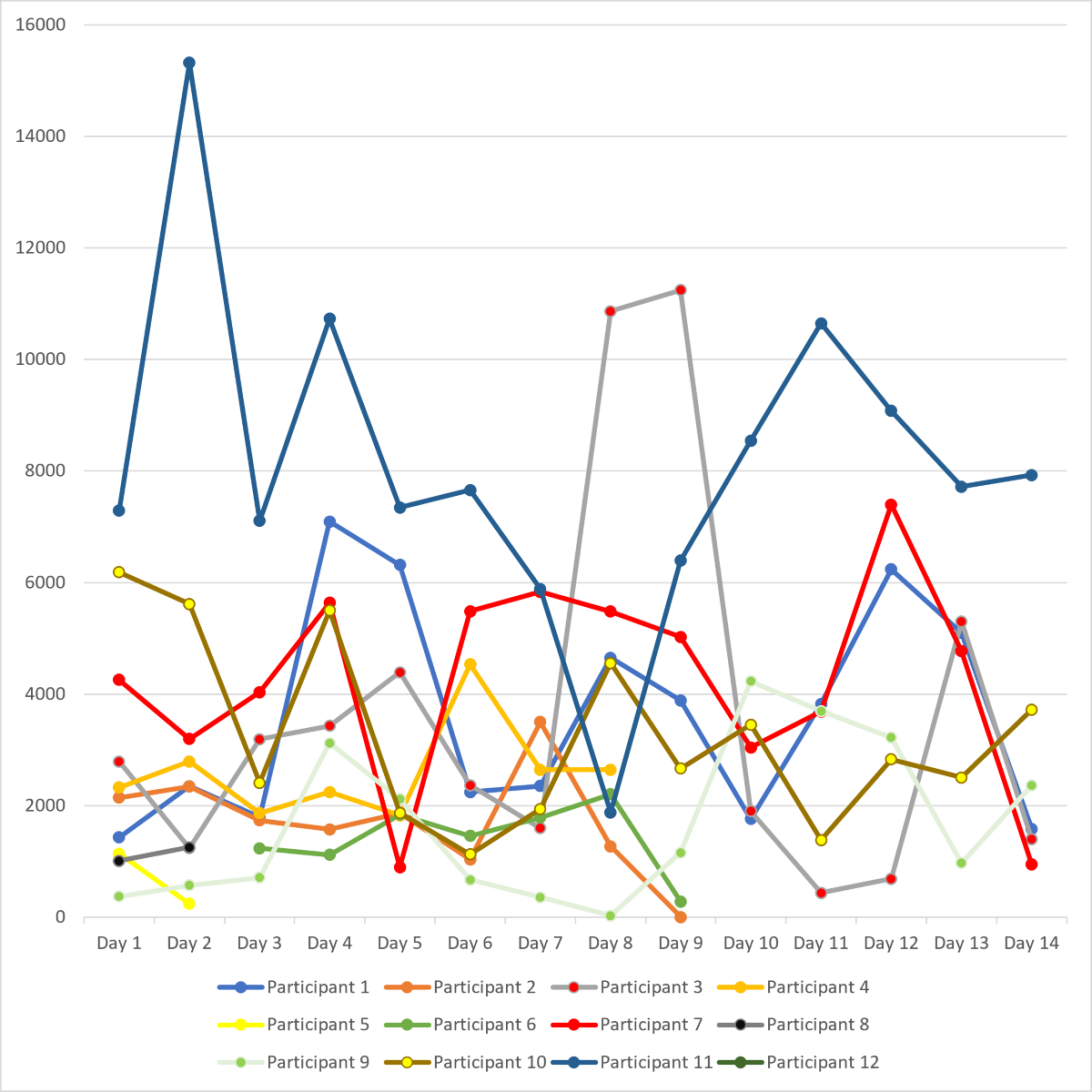
Daily step counts during the 2-week walking challenge.

Figure 1 displays the daily step counts of individual participants who synchronized their steps with the 50K4Life app over the 2-week duration of the walking challenge. It should be noted that most participants had daily step counts <7000 steps/day.

## Discussion

### Overview

User experience and preference can influence participants’ engagement of app-delivered physical activity promotion programs [13,21]. The purpose of this beta test in the 50K4Life study was to assess the usability and acceptability of the 50K4Life app designed to deliver an evidence-based walking challenge to public school employees [44,49]. The 2 main research questions guiding our work were whether the app had the features and functions to deliver the intervention as planned, and how feasible and acceptable the app was to the participants. As anticipated, participants appreciated certain aspects of the app, while identifying other aspects they wanted improved, despite efforts to follow evidence-based mHealth promotion recommendations [49,50]. Participants were generally satisfied with the app and felt it was usable. However, participants found the app unengaging and unattractive and had issues with syncing and setup. Overall, the findings are consistent with published studies testing the usability and acceptability of mHealth apps in community-based health promotion programs [41,51].

### Principal Results

Overall, participants demonstrated high engagement with the 50K4Life app features, though their satisfaction with some of them was mixed. In this study, participants appreciated the app’s simplicity and ease of use. Reminders were viewed favorably. While being able to sync with multiple devices was viewed positively, participants voiced difficulties in synchronization of step counts and a need for additional support to use the app. Most synchronization difficulties reported by participants stemmed from third-party integrations (Fitbit, Google Fit, and Apple Health), where correct user setup (permissions, account linking, and device syncing) is required. Misconfigurations on the participants’ side are a well-documented source of error in mHealth studies [52]. At the time of the beta test, our monitoring was limited to client-side reports, and we did not systematically capture application programming interface success or failure rates on the server. This limited our ability to provide independent verification of the Pathverse platform’s backend reliability for the study period. In the future, we plan to implement server-side monitoring to track application programming interface request success rates, error codes, and latency. This will allow us in future studies to differentiate more clearly between user-side misconfigurations, third-party service constraints, and Pathverse platform reliability. In addition, we will provide a user tutorial on the features and functions to familiarize the participants with the features and functions in the app as suggested by the participants [52].

The delivery of multiple components of the beta test was generally found to be feasible. Participants engaged in the walking challenge, tracking and syncing their own steps and those of their teammates. This enabled them to monitor their teammates and other school districts’ teams on the leaderboard. However, participants’ engagement in the interactive educational sessions was low due to a short window. Notification reminders of upcoming lessons would have increased the open rates of the lessons [1].

The beta test and evaluation generated useful feedback for improving the app for subsequent implementations. This beta test is part of a larger study designed to increase walking among school employees. Recruiting participants from local school district administrative offices who had a first-hand understanding of school employees’ daily life reflects the community-based approach to the overall 50K4Life study. Moreover, input from the community advisory board and participants in the beta test helped shape the app’s content and tailor the design to the target community’s needs and interests.

Despite having extensive preparation meetings leading up to the beta test, it was evident that unforeseen technicalities hindered the study’s execution as planned [1]. Consequently, during the beta test, we encountered several challenges. During implementation, we found that while Pathverse provided a user-friendly platform for the 50K4Life app, it did not allow communications with the participants regarding their engagement in the beta test activities. For instance, we required the ability to send automated messages to participants based on their progress in the walking challenge, that is, did they meet their daily goal. This problem can be resolved by creating an algorithm-based feature to track daily step counts in the app and provide updates on goal achievement in a timely manner [53]. Participant feedback will allow us to work with the Pathverse development team to develop new features and functions that will improve user experience and engagement in the intervention.

### Strengths and Limitations

User experience testing is intended to be an iterative process, sometimes with multiple rounds of testing and refinement [53]. A major strength of the app is that the content is based on findings of ongoing walking challenges in the El Paso community, providing a theory-driven motivational approach with tailoring for the setting [37]. Additionally, the beta test followed the IDEAS framework to garner acceptability and usability from a sample similar to the intended 50K4Life population, informing the design and testing of the app to meet future users’ needs [43]. The limitations of the study included a short period that prevented an in-depth evaluation of the participant experience and the app’s features and functions. A second limitation is that there were no beta test participants who selected the Spanish assessments. It is possible that participants who prefer Spanish may experience the app features differently, and that this input was not captured as part of the beta test. Issues in synchronization with the 50K4Life app was another limitation that might have influenced the participant’s engagement in the pilot study. Finally, some of the issues identified were beyond the scope of the beta test. However, we will take the participants’ suggestions in the refinement of the app and evaluate their impact in future implementations of the app [54]. Finally, we did not conduct a cost or time analysis at this phase of the study. Having that information would improve our understanding of the implementation feasibility of the 50K4Life app.

### Future Research

The next logical step in the development of the 50K4Life app is to address the constructive feedback provided by beta test participants and then evaluate the app again in the pilot study. This is part of the planned pilot study where additional participant comments will be used to further refine the user experience. In this future study, we will investigate cultural or linguistic barriers to accessing the app for Spanish-speaking staff. Additionally, we will be conducting a cost-effectiveness evaluation to determine operational costs that would have implications for real-world adoption and scalability. This beta study represents the first step in implementation, and participant feedback will inform future revisions to the app preceding the planned full-scale trial [55].

### Conclusions

The use of the IDEAS framework as a community-based participatory approach in the design of the 50K4Life app contributed to the enhancement of the functionality and acceptability in target communities with high chronic disease burden and increased the potential of success in “real-world” use. Through the 50K4Life app beta test, we gained valuable knowledge on barriers that need to be addressed so that we may optimize our use in the full-scale trial. Lessons learned from the beta test can inform future dissemination efforts of mHealth interventions in different settings for health promotion.

## Data Availability

Data access requests from this study can be made directly to the corresponding author.

## Abbreviations

IDEAS: Integrate, Design, Assess, and Share
mHealth: mobile health
REDCap: Research Electronic Data Capture
